# Simulation-Based Outreach Program to Support Community Hospitals’ Team Readiness for Obstetric Emergencies Across a Rural Health System

**DOI:** 10.7759/cureus.117482

**Published:** 2026-10-05

**Authors:** Jesse M Rideout, Micheline Chipman, Beth Gray, Rebecca Hunt, Emily Watson, Sarah Austin, Anya Cutler, Lauren Weller, Jeffrey A Holmes, Leah Mallory

**Affiliations:** 1 Emergency Medicine, MaineHealth Maine Medical Center, Portland, USA; 2 Emergency Medicine, Tufts University, Boston, USA; 3 Medical Education and Simulation, MaineHealth Maine Medical Center, Portland, USA; 4 Obstetrics and Gynecology, MaineHealth Maine Medical Center, Portland, USA; 5 Obstetrics and Gynecology, Tufts University, Boston, USA; 6 Center for Outcomes Research and Evaluation, MaineHealth Maine Medical Center, Portland, USA; 7 Medical Education and Simulation, Tufts University, Boston, USA; 8 Pediatrics, MaineHealth Maine Medical Center, Portland, USA; 9 Pediatrics, Tufts University, Boston, USA

**Keywords:** emergency obstetric care, healthcare simulation, interprofessional team training, maternal safety, obstetric complications, rural health settings, rural health systems, simulation in medical eduction

## Abstract

Background

Rural hospitals face persistent challenges in sustaining readiness for obstetric emergencies due to declining birth volumes, workforce shortages, and limited opportunities for hands-on practice. The Maine Obstetrical Medical Simulation (MOMSim) program was developed to support interprofessional team preparedness across a rural health system. This study evaluated program implementation and its impact on participants’ confidence in managing obstetric emergencies.

Methodology

An in situ, simulation-based obstetric emergency training program was delivered across eight community hospitals within a rural health system from 2022 to 2025. Each site received two half-day training visits approximately one year apart. The curriculum incorporated targeted skills stations, high-fidelity in situ simulations, and structured debriefings. Participant demographic information was collected, and anonymous pre- and post-training surveys assessed confidence across multiple domains on a five-point Likert scale. Linear mixed-effects models evaluated aggregate pre- and post-training changes across sites and visits and differences in baseline confidence between the first and second visit; confidence was also compared across hospital sites and professions.

Results

Across 16 training sessions, 287 interprofessional participants engaged in the program. Confidence increased significantly across all assessed domains following training, with mean improvements ranging from 0.24 (95% confidence interval (CI) = 0.11-0.37) to 0.51 (95% CI = 0.35-0.68) Likert points. No significant differences in baseline confidence were observed between visits. Pre-training comparisons between sites identified significant differences in perceived resources, team confidence, and team cohesion.

Conclusions

The MOMSim program was successfully implemented across a geographically dispersed rural health system and was associated with significant improvements in interprofessional team confidence. Systemwide simulation outreach may provide a feasible approach to strengthening obstetric emergency preparedness and team-based readiness of low-volume rural delivery units.

## Introduction

One in three U.S. counties has no birthing facilities or obstetric clinicians, affecting 2.4 million women of reproductive age and 149,000 infants born each year [1]. Nationwide, over 500 hospital obstetric units have closed in the last decade, worsening areas of “maternity care deserts” [2,3]. These closures are driven by multiple factors, most notably low birth volumes [4], along with decreasing reimbursement and unfavorable payer mix, high costs of obstetric care, workforce shortages, demographic shifts, and hospital consolidations [5-7].

Maine is considered the most rural state in the United States, with over half of its land area uninhabited and more than 60% of the population living in rural regions [8]. Between 2014 and 2023, eight birthing units in Maine have closed, and in 2025 alone, four additional units closed, leaving just 17 hospitals statewide with active delivery services [9,10].

Widespread closures of obstetric units in Maine and nationwide, along with low birth volumes seen at many remaining rural and smaller community hospitals, directly limit opportunities for clinical exposure and coordinated team responses to high-risk obstetric emergencies. Procedural competencies required to manage obstetric emergencies, including shoulder dystocia maneuvers, postpartum hemorrhage management, and neonatal resuscitation, deteriorate over time because of skill decay [11-13]. Low-volume hospitals have been shown to experience higher rates of maternal complications, including postpartum hemorrhage and unplanned cesarean sections [14,15]. Furthermore, rural communities that have lost hospital-based obstetric units experience significant increases in out-of-hospital and preterm births, which are associated with increased risk of neonatal complications [16].

Simulation training is a critical component of obstetric emergency preparedness and team performance. The Joint Commission recognizes simulation and multidisciplinary drills as essential strategies to improve maternal safety and reduce preventable morbidity and mortality. In its 2019 R3 Report on Maternal Safety Standards, the Joint Commission explicitly recommended that hospitals conduct obstetric emergency drills at least annually, including for maternal hemorrhage and severe hypertension/preeclampsia, to identify system issues, enhance interprofessional coordination, and support continuous quality improvement efforts [17].

Brogaard et al. (2022) synthesized 21 studies and found that obstetric team training, most commonly simulation-based, was associated with reductions in severe maternal and neonatal outcomes, including postpartum hemorrhage severity and brachial plexus injury. However, the overall certainty of evidence remains moderate [18].

Developing and sustaining a simulation-based training outreach program in Maine presents distinct challenges because remote regions and critical access hospitals are dispersed across a broad geographic footprint. In addition, the distribution of obstetric providers and services varies substantially statewide, further complicating travel logistics. Rural hospitals also face workforce capacity and retention challenges that limit local educational infrastructure and simulation resources [19], while competing clinical demands can make it difficult for staff to secure protected time to participate in simulation-based training.

The Maine Obstetrical Medical Simulation (MOMSim) program was developed as an in situ simulation-based training initiative to address the need for obstetric team emergency readiness across eight community hospitals. Our primary objectives were to successfully implement the program across our health system and to determine whether participant confidence increased following in situ simulation training. Successful implementation was defined as the development of a standardized curriculum, delivery of the training at all eight participating hospitals, and completion of a repeat training visit approximately one year later. Secondary objectives included comparing participant confidence between visits, sites, and health professions.

## Materials and methods

We implemented an in situ simulation training program with two visits per site across our rural health system between March 2022 and September 2025 and conducted quasi-experimental pre- and post-training surveys assessing obstetric team participants’ confidence in managing obstetric emergencies. The MaineHealth Institutional Review Board determined this program did not constitute human subject research.

Context

The MaineHealth system includes Maine Medical Center-Portland (MMC-P), a 744-bed urban academic center, and, at the time of the study, eight community hospitals with labor and delivery (L&D) services located across Maine and Eastern New Hampshire. MMC-P has approximately 3,592 deliveries annually; each of the community hospitals included in the program has fewer than 550 deliveries per year. The intervention was delivered at all eight community hospitals within the MaineHealth system. Rural status of participating hospitals was determined using the Health Resources and Services Administration (HRSA) Rural Health Grants Eligibility Analyzer, which defines rural areas based on a combination of non-metropolitan county status and Rural-Urban Commuting Area (RUCA) codes [20]. Using this definition, seven out of eight participating community hospitals were located in HRSA-designated rural areas. In 2024, one of our participating critical access hospitals closed its L&D unit (Table 1).

**Table 1:** Participating hospital demographics. ^*^: Rural area and population density are based on the county in which the hospital is located. ^¥^: The US poverty rate is 10.6% (2024). ^Ω^: Defined by the Health Resources and Services Administration Rural Health Grants Eligibility Analyzer.

Hospital	A	B	C	D	E	F	G	H
Rural area^*^	93.50%	99.80%	100%	99.2%	84.60%	97.50%	99.70%	99.60%
Population density (sq mi)^*^	208.0	17.6	75.7	54.4	352.0	109.0	27.8	54.4
Poverty rate^¥^	8.10%	12.90%	8.80%	7.90%	8.60%	10.20%	13.00%	11.60%
Rural^Ω^	No	Yes	Yes	Yes	Yes	Yes	Yes	Yes
Critical access hospital?	No	No	Yes	Yes	No	No	Yes	Yes
Live births per year, mean	450	230	100	225	510	215	150	125

The Hannaford Center for Safety, Innovation and Simulation (the Sim Center) was established in 2010. The Sim Center is an 18,000 square foot simulation space in Portland, Maine, located less than two miles from MMC-P and more than 100 miles from the most distant community hospital within the system. Programs include mannequin-based simulation with a wide array of skills trainers and patient simulators, a mobile simulation unit, a growing virtual and augmented reality simulation program, and a human simulation program that includes more than 60 trained standardized patients. In 2019, with the unification of the health system, the Sim Center was asked to extend services across MaineHealth.

In 2015, the Sim Center partnered with obstetric physician and nurse educators at MMC-P to initiate a quarterly, in situ, interprofessional team training program. Initially focused on postpartum hemorrhage, the program has evolved to address a variety of emergencies, including code team responses on obstetric units, sepsis, hypertensive emergencies, and more. These quarterly simulations now routinely include 50-100 participants (in person and remote) from multiple disciplines (e.g., obstetrics, anesthesiology, family medicine) and professions, including nurses, physicians, advanced practice providers, respiratory therapists, pharmacists, and clinical nurse assistants. In 2019, following system integration, expansion of simulation-based team training for obstetric emergencies across the health system was identified as a top priority. The MOMSim program was developed with foundational and philanthropic support.

Participants

Participants included clinical obstetric team members from rural hospitals, including obstetricians, nurse-midwives, L&D nurses, and clinical nursing assistants. Recruitment was coordinated collaboratively by the obstetric Sim team (see below) and local hospital leadership during a series of planning meetings for each training visit. Potential participants were identified based on their roles in obstetric emergency care, with recruitment and scheduling facilitated through email to develop a participant list before each session.

Program description

The obstetric Sim team includes clinical educators from the Sim Center (MC and BG), an obstetrician educator (RH), and perinatal educators (EW and SA). Community agreements to participate were signed by hospital and obstetric unit leadership at each site, pledging support for team attendance at two half-day simulation training events occurring approximately one year apart. Local nurse and physician collaborator leads were identified at each site; these site leaders participated in planning and producing the event and attended a post-event virtual follow-up meeting after each training. Training days included one full or two half-day sessions, depending on the size of local teams.

Learning objectives for the curriculum focused on recognition and management of high-risk obstetric emergencies, including postpartum hemorrhage, hypertensive emergencies, and shoulder dystocia, as well as effective interprofessional teamwork. Skills stations, simulation scenarios, and facilitated debriefings provided formative opportunities to practice and receive feedback on these objectives. The obstetric Sim team traveled to each hospital with mannequins and simulation equipment for training days. Each four-hour training began with pre-briefing, followed by skills stations focused on quantification of blood loss, medication administration for hypertensive emergencies, management of shoulder dystocia, and use of intrauterine hemorrhage management devices. Skills sessions were followed by three high-fidelity scenarios (postpartum hemorrhage, hypertensive emergency, and shoulder dystocia) produced in situ in an L&D suite and broadcast to a conference room. Participants were divided into interprofessional teams, ensuring that everyone actively engaged in at least one simulation. All participants viewed the simulation remotely, and a structured debriefing occurred immediately following each scenario in the conference room. Debriefings were co-facilitated by a clinical nurse educator (MC) and an OB educator (RH). Debriefings followed the PEARLS framework [21] and included identification of latent safety threats and system improvement opportunities [22]. Identification, mitigation, and retesting of latent safety threats through the systems testing component of the MOMSim program have been reported separately [23].

A follow-up virtual meeting with local site leaders was held one to two months after the training to revisit action items identified during training and plan next steps. Each hospital participated in two training days approximately one year apart.

Evaluation

Pre- and post-training anonymous surveys were administered at each of the two training visits at all participating hospitals. Surveys captured basic demographic information and self-reported confidence in managing obstetric emergencies across multiple confidence domains, rated using a five-point Likert scale (1 = no confidence and 5 = highly confident/fully prepared). Self-reported confidence across the assessed domains was the educational outcome of interest; no single confidence domain was designated as the primary outcome. The survey was adapted from a previously published confidence assessment used in a rural neonatal resuscitation simulation program [24] and modified by the investigator team, which included experts in obstetric emergencies and simulation-based education, to reflect the objectives and clinical content of the current program. The pre-training survey was distributed approximately two weeks before each training, with participants reminded upon arrival to complete it if they had not already done so. An analogous post-training survey was administered at the conclusion of the session, with a follow-up email sent two weeks later to encourage completion. The complete survey instrument, including the wording of individual items, is provided in the Appendices. Survey questions with missing responses were excluded from the tables and figures; missing data ranged from zero to four participants per survey question. All surveys and data management were conducted in REDCap [25,26].

Analysis

A linear mixed-effects model was used with confidence scores (1-5 scale) as the outcome. The primary predictor was pre- versus post-training status across training visits, with an additional comparison of pre-training confidence between the first and second visit. The model included random effects for visit number and for hospital site to account for clustering of observations within sites and visits. Model assumptions were assessed and met and included: linearity, homoscedasticity, influential observations, and normality of residuals and random effects. Because we did not have participant identifiers, within-individual correlation could not be assessed. Group differences in confidence across sites and professions were analyzed using Wilcoxon rank-sum tests. All analyses were conducted in R version 4.2.1. The lme4 package was used to run linear mixed-effects models, and the gtsummary package was used to compare confidence between sites and run Kruskal-Wallis rank-sum tests.

## Results

A total of 16 half-day training sessions were conducted, involving 287 interprofessional L&D team members across participating sites (Table 2). Half-day sessions included between nine and 26 participants each.

**Table 2:** Description of participants across community hospital sites. ^1^: Registered nurse. ^2^: Clinical nurse midwife. ^3^: Certified nursing assistant; licensed nursing assistant. ^4^: Other participant types: ʷ: interprofessional staff (e.g., respiratory therapy, pharmacy); ˣ: medical student; ʸ: student nurse midwife; ᶻ: education lead/simulation educator.

Site	Visit #	RN^1^	CNM^2^	Physician	CNA/LNA^3^	Other^4^	Total
A	1	13	-	5	4	-	22
	2	9	-	8	5	1^x^	23
B	1	15	-	3	-	-	18
	2	16	1	4	-	-	21
C	1	9	2	1	-	2ᶻ	14
	2	9	2	3	-	-	14
D	1	15	2	2	2	1ʷ	22
	2	12	2	3	2	-	19
E	1	13	5	6	-	2ˣ	26
	2	19	3	3	-	1^x^	26
F	1	8	3	1	-	1ʸ	13
	2	12	5	1	-	-	18
G	1	7	-	2	-	-	9
	2	10	-	3	-	-	13
H	1	11	3	2	-	-	16
	2	8	3	2	-	-	13

Differences in confidence by visit and site

Self-rated confidence across all assessed domains increased significantly following training in the combined analysis of both training visits (Table 3). There were no differences in pre-training confidence across domains between visits one and two (Table 4).

**Table 3:** Average change in Likert confidence ratings between pre- and post-trainings across sites. ^1^: The total N counts both the pre- and post-survey for a single participant. ^2^: Pre-training is the reference category. ^3^: CI = confidence interval. Beta estimates are the average change in Likert scale points between pre- and post-surveys, with pre-survey as the reference category, controlling for intra-site and intra-visit correlation. For example, on average, participants rated confidence in their knowledge of obstetrical emergencies 0.51 points higher in the post-survey compared to the pre-survey (95% CI = 0.35, 0.68; p < 0.001).

Confidence domain	N^1^	Beta^2^ (95% CI^3^)	P-value	Wald statistic
Knowledge	448	0.51 (0.35–0.68)	<0.001	6.07
Equipment	448	0.46 (0.30–0.63)	<0.001	5.51
High‑risk deliveries	448	0.31 (0.14–0.47)	<0.001	3.71
Medications	442	0.41 (0.23–0.58)	<0.001	4.62
Intravenous access	442	0.48 (0.23–0.73)	<0.001	3.79
Team leader	449	0.27 (0.11–0.43)	0.001	3.31
Resources	448	0.43 (0.27–0.59)	<0.001	5.29
Team confidence	450	0.48 (0.34–0.63)	<0.001	6.53
Communication	450	0.24 (0.11–0.37)	<0.001	3.62
Team cohesion	448	0.42 (0.26–0.58)	<0.001	5.25

**Table 4:** Average change in Likert confidence ratings before each session between visit one and two across the sites. ^1^: Visit 1 is the reference category. ^2^: CI = confidence interval. Beta estimates are the average change in Likert scale points between pre-training surveys of visit 1 and visit 2, with visit 1 as the reference category, controlling for intra-site and intra-visit correlation.

Confidence domain	N	Beta^1^	95% CI^2^	P-value	Wald statistic
Knowledge	290	0.03	-0.18, 0.24	0.8	0.274
Equipment	288	0.06	-0.16, 0.27	0.6	0.528
High-risk deliveries	288	0.00	-0.22, 0.21	>0.9	-0.021
Medications	286	-0.02	-0.24, 0.20	0.9	-0.185
Intravenous access	286	-0.26	-0.57, 0.04	0.092	-1.69
Team leader	290	-0.08	-0.28, 0.12	0.4	-0.794
Resources	288	0.01	-0.18, 0.21	0.9	0.140
Team confidence	290	0.05	-0.14, 0.23	0.6	0.506
Communication	290	0.01	-0.16, 0.17	>0.9	0.093
Team cohesion	288	0.05	-0.14, 0.25	0.6	0.549

Comparison of pre-training confidence across sites revealed significant differences in perception of resources, team confidence, and team cohesion (Table 5).

**Table 5:** Comparison of confidence across sites according to pre-training surveys. ^1^: Median (interquartile range). ^2^: Kruskal-Wallis rank-sum test.

Confidence domain	A (N= 43^1^)	B (N = 38^1^)	C (N = 31^1^)	D (N = 33^1^)	E (N = 56^1^)	F (N = 31^1^)	G (N = 23^1^)	H (N = 35^1^)	P-value^2^	Χ^2^ (df = 7)
Knowledge	4.00 (4.00, 5.00)	4.00 (4.00, 4.00)	4.00 (3.00, 5.00)	4.00 (3.00, 4.00)	4.00 (3.00, 4.25)	4.00 (3.50, 4.00)	4.00 (3.00, 5.00)	4.00 (4.00, 5.00)	0.2	10.51
Equipment	4.00 (4.00, 5.00)	4.00 (4.00, 4.00)	4.00 (3.00, 4.00)	4.00 (3.00, 5.00)	4.00 (3.00, 4.00)	4.00 (3.00, 5.00)	4.00 (3.50, 5.00)	4.00 (4.00, 5.00)	0.060	13.54
High-risk deliveries	5.00 (4.00, 5.00)	5.00 (4.00, 5.00)	4.00 (3.50, 5.00)	4.00 (4.00, 5.00)	4.00 (4.00, 5.00)	4.00 (4.00, 5.00)	4.00 (3.50, 5.00)	4.00 (4.00, 5.00)	0.3	7.94
Medications	4.00 (4.00, 5.00)	4.00 (4.00, 5.00)	4.00 (3.00, 5.00)	4.00 (4.00, 5.00)	4.00 (4.00, 5.00)	4.00 (3.00, 4.00)	4.00 (3.50, 5.00)	4.00 (3.00, 5.00)	0.5	6.69
Intravenous access	4.00 (2.00, 5.00)	4.00 (3.00, 5.00)	3.00 (2.00, 4.50)	4.00 (2.00, 5.00)	3.00 (2.00, 4.00)	3.00 (2.00, 4.00)	4.00 (2.50, 4.00)	4.00 (2.00, 5.00)	0.4	7.42
Team leader	5.00 (3.00, 5.00)	5.00 (4.00, 5.00)	4.00 (3.00, 5.00)	5.00 (4.00, 5.00)	4.00 (4.00, 5.00)	4.00 (4.00, 5.00)	5.00 (4.00, 5.00)	4.00 (4.00, 5.00)	0.10	12.00
Resources	4.00 (4.00, 5.00)	4.00 (4.00, 5.00)	3.00 (3.00, 4.00)	4.00 (4.00, 5.00)	4.00 (3.00, 4.00)	4.00 (3.00, 4.00)	4.00 (3.50, 4.50)	4.00 (3.00, 4.00)	<0.001	32.74
Team confidence	4.00 (4.00, 5.00)	4.00 (4.00, 5.00)	3.00 (3.00, 4.00)	4.00 (4.00, 5.00)	4.00 (3.00, 4.00)	4.00 (3.00, 4.00)	4.00 (4.00, 4.50)	4.00 (3.00, 4.00)	<0.001	38.39
Communication	5.00 (4.00, 5.00)	5.00 (4.00, 5.00)	5.00 (4.00, 5.00)	5.00 (4.00, 5.00)	4.00 (4.00, 5.00)	4.00 (4.00, 5.00)	5.00 (4.00, 5.00)	4.00 (4.00, 5.00)	0.076	12.86
Team cohesion	4.50 (4.00, 5.00)	4.00 (4.00, 5.00)	3.00 (3.00, 4.00)	4.00 (4.00, 5.00)	4.00 (3.00, 4.00)	4.00 (3.00, 4.00)	4.00 (4.00, 5.00)	4.00 (3.00, 4.00)	<0.001	32.12

Differences in confidence by role

Confidence differed significantly by role in all categories except resources and communication. Obstetric physicians reported the highest confidence in their knowledge of obstetric emergencies (p < 0.0001; χ²= 49.93; df = 3). Registered nurses reported the highest confidence in their ability to locate medications and equipment (p < 0.0001; χ² =18.3; df = 3). Midwives and obstetric physicians reported the highest confidence in managing high-risk deliveries (p < 0.0001; χ² = 50.46; df = 3) and medications (p < 0.0001; χ² = 47.04; df = 3). Registered nurses had the highest confidence in performing emergency intravenous access (p < 0.0001; χ²= 84.84; df = 3). Obstetric physicians were most confident in identifying a leader (p <0.0001; χ²= 14.07; df = 3). Confidence in available resources was high across all roles and did not differ significantly (p = 0.5; χ²= 2.51; df = 3), and confidence in effective communication was similarly high without significant differences by role (p = 0.065; χ² = 7.23; df = 3) (Figure 1).

**Figure 1:**
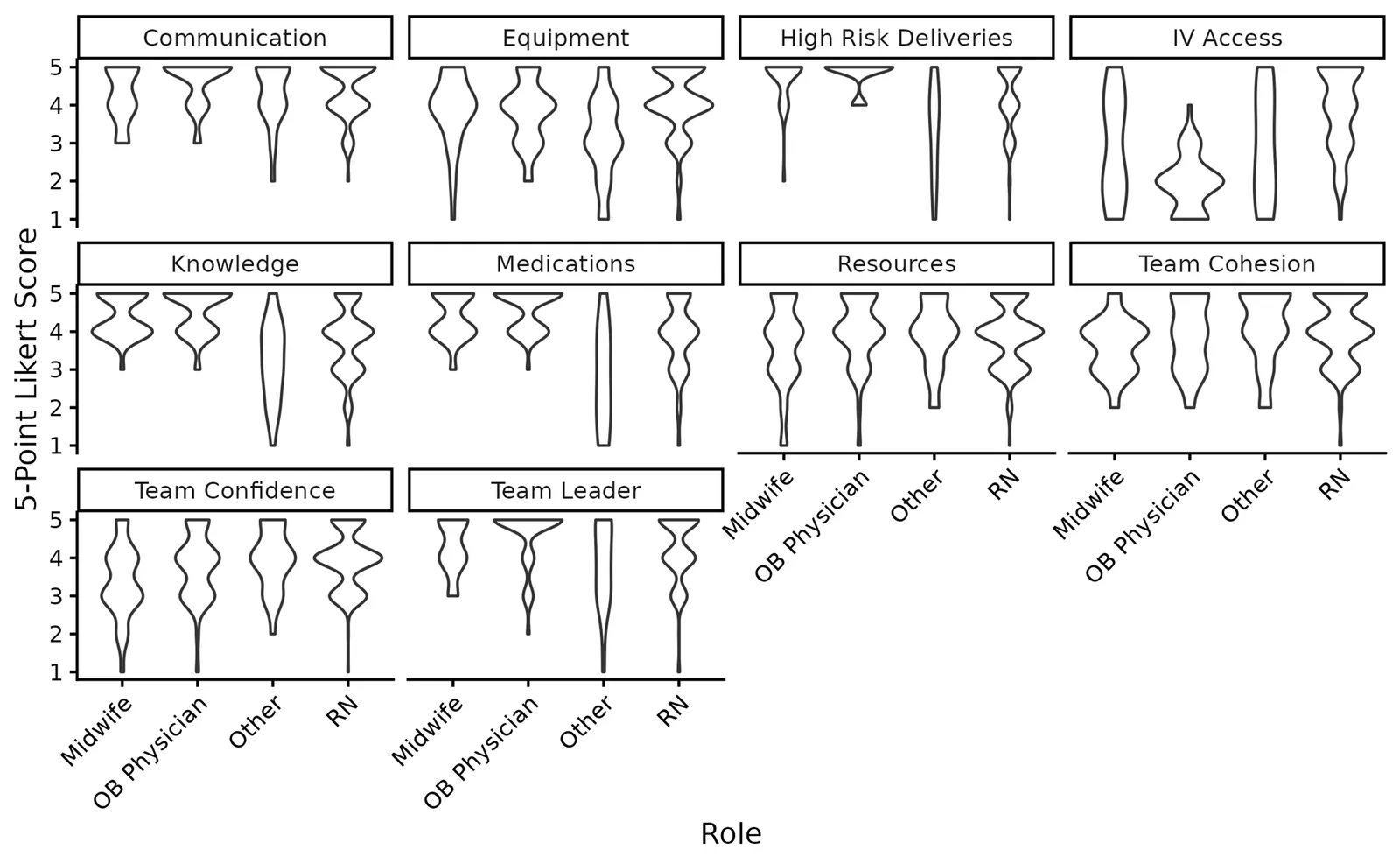
Confidence by roles in pre-training surveys. IV = intravenous; OB = obstetric; RN = registered nurse

## Discussion

We successfully implemented an interprofessional in situ obstetric emergency simulation program across eight hospitals in our geographically dispersed rural health system, achieving our implementation goals of developing a standardized curriculum, delivering the program to all participating hospitals, and completing a repeat training visit approximately one year later. Over four years, 287 team members participated in the program, which focused on emergency management skills, teamwork, and resource utilization. On average, 18 participants attended each session, reflecting strong multidisciplinary engagement. Confidence improved significantly across all evaluated domains following training. Pre-training confidence did not differ significantly between visit one and two across assessed domains. Pre-training comparisons across hospitals showed no significant differences in most domains, with the exception of team confidence, team cohesion, and resources. These variations may reflect differences in team familiarity and local resource availability and highlight opportunities for system-wide simulation initiatives to promote greater equity across sites.

Our findings add to a growing body of evidence supporting simulation-based approaches to obstetric emergency preparedness across rural and community hospitals. Several states have implemented statewide or regional obstetric simulation programs to strengthen maternal safety and emergency preparedness, particularly in rural or low-volume hospitals. Models such as Iowa’s mobile obstetric simulation initiative delivered train-the-trainer workshops and in situ drills to rural hospitals and emergency departments, with participants reporting improved knowledge and skills and strong feasibility for ongoing deployment [27]. Beyond traditional onsite teams, simulation programs integrated into tele-obstetric workflows across multiple rural and community hospitals have also been shown to improve team confidence in managing obstetric emergencies and using tele-obstetric processes, supporting scalable educational models for geographically dispersed healthcare systems [28]. These programs report high participant satisfaction, improved clinical confidence, and enhanced team performance across disciplines, especially for managing postpartum hemorrhage and hypertensive emergencies. Together with our findings, these programs support the feasibility of systemwide obstetric simulation and its potential to improve confidence and readiness across rural and community hospitals.

The MOMSim training initiative fostered relationships between simulation educators and local obstetric teams, introduced clinicians to simulation-based learning, and enhanced team member confidence in obstetric emergency management. Beyond measurable improvements in confidence, the program established a foundation for ongoing simulation efforts within our health system. These findings align with the American College of Obstetricians and Gynecologists (ACOG) 2018 Position Statement [29], which emphasizes that rural and low-volume settings often face challenges in maintaining clinical skills due to limited exposure and opportunities for practice. ACOG further notes that rural clinicians frequently work in relative isolation with restricted access to consultation and support services. Similarly, the National Rural Health Association’s 2024 white paper, Maternal Health in Rural America, highlights that rural obstetric and family physicians experience professional isolation, unpredictable on-call demands, long travel times, and reduced peer support, factors associated with lower career satisfaction [30]. These challenges underscore the potential value of accessible, recurring training opportunities for rural obstetric teams. The MOMSim program laid the groundwork for ongoing, regular obstetric simulation training, currently occurring within our system.

Limitations

Limitations of this study include the use of de-identified surveys, which prevented determination of how many participants completed both the first and second training visits versus a single session and precluded the ability to determine pre/post confidence changes at the individual level. Consequently, comparisons of pre-training confidence between visit one and two reflect group-level differences and cannot be interpreted as longitudinal changes in confidence among individual participants. This approach supported voluntary involvement and accommodated the realities of busy clinical schedules. The study did not include a comparison group, and outcomes were assessed immediately following training, limiting conclusions regarding retention or longer-term effects. Participation may have introduced selection bias, while incomplete survey response may have introduced nonresponse bias. Confidence was measured using a locally developed self-report instrument without formal validity evidence. We also did not incorporate objective outcome measures, such as knowledge assessments, procedural skill scoring, or structured team performance evaluations for the high-fidelity simulation cases. Although team behaviors and adherence to ACOG-recommended practices were addressed formatively during debriefing, data were not systematically captured for analysis. This approach was intentional to ensure that the training emphasized psychological safety and prioritized reflective debriefing and reinforcement of ACOG clinical guidelines and team-training best practices rather than summative performance assessment. Statewide travel and transport of simulation equipment and personnel are costly and likely not sustainable at all sites with the frequency needed to maintain skills and confidence. However, the acceptability and success of this MOMSim program have led to a follow-up program that emphasizes sustainability and frequent longitudinal training, using lower-technology simulation equipment with online curricular resources and tele-simulation support from the Sim Center.

However, the acceptability and success of this MOMSim program have led to a follow-up program that emphasizes sustainability and frequent longitudinal training, using lower-technology simulation equipment with online curricular resources and tele-simulation support from the Sim Center (Abstract: Gray B, Chipman M, Weller L, Watson E, Austin S, Palniker T, Mallory L. Implementation of MOMSim 2.0. Costas T. Lambrew Research Retreat; 2025).

## Conclusions

The MOMSim program was successfully implemented across a geographically dispersed rural health system and was associated with significant improvements in interprofessional and team confidence in obstetric emergency management. Beyond its educational benefits, this program may enhance healthcare team cohesion by connecting clinicians with simulation educators and obstetric content experts through simulation-based learning. These relationships may be particularly valuable for clinicians in more remote rural areas where opportunities for collaboration and professional development are more limited. Simulation outreach provides a feasible approach to obstetric emergency training in low birth volume settings. Building on this initial program implementation, a subsequent initiative was developed and implemented to provide more frequent, sustainable obstetric simulation training using low-technology obstetric training kits, educational resources, and telesimulation support for birth unit teams throughout our rural health system.

## Figures and Tables

**Figure 2:**
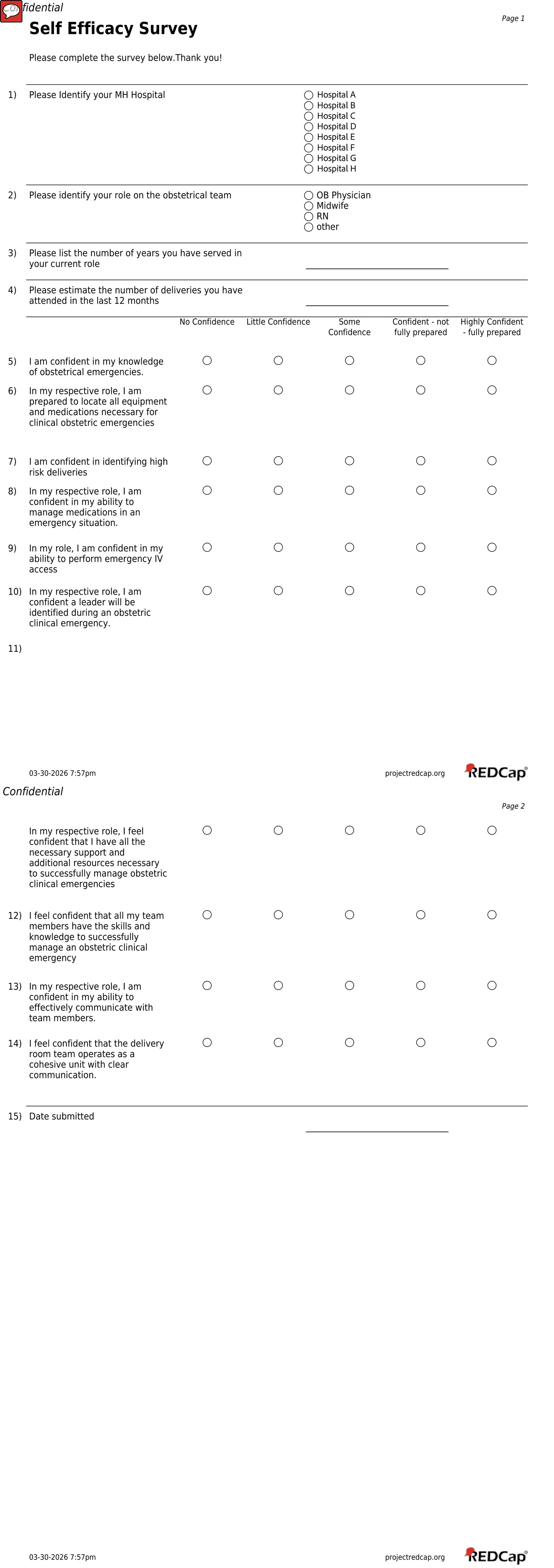
Maine Obstetrical Medical Simulation (MOMSim) obstetric emergency confidence survey: pre and post.
